# AI Ethics Guidelines: Time to Include Animals

**DOI:** 10.1007/s11948-025-00578-5

**Published:** 2026-01-08

**Authors:** Simon Coghlan, Christine Parker, Reeva Lederman

**Affiliations:** 1 School of Computing and Information Systems, Centre for AI and Digital Ethics, The University of Melbourne, Australian Research Council Centre of Excellence for Automated Decision Making and Society, Melbourne, Victoria, Australia; 2 Melbourne Law School, The University of Melbourne and Australian Research Council Centre of Excellence for Automated Decision Making and Society, Melbourne, Victoria, Australia; 3 School of Computing and Information Systems, The University of Melbourne, Melbourne, Victoria, Australia

**Keywords:** Nonhuman animals, Artificial intelligence, Sentience, Ethics guidelines, Moral value

## Abstract

Artificial intelligence (AI) ethics guidelines make strong appeals for ensuring that AI benefits human beings without unjustifiably harming them. Yet these guidelines largely overlook AIs ethically relevant positive and negative impacts on sentient nonhuman animals. This paper argues that it is time to include sentient animals in AI ethics guidelines produced by governments, supranational and intergovernmental bodies, NGOs, academics, professional groups, for-profit corporations, and the like. AI ethics guidelines should explicitly and adequately include values and principles that protect and promote sentient animal interests. The case proceeds by considering five possible objections to recognising animals in these guidelines: 1) Animals and their interests are not significantly affected by AI; 2) Humans lack duties to protect or help animals; 3) Considering animals is overridden by more pressing duties to humans; 4) There is too much disagreement about how animals should be treated; 5) AI ethics guidelines are unhelpful or counterproductive. These objections, the paper argues, ultimately fail. A very important reason for being more inclusive of animals, it claims, is their ongoing moral and social invisibility. Paying more attention to sentient animals as morally significant interest-holders in the rapidly evolving AI ethics discourse would go one step towards redressing this problem of marginalisation. Finally, this paper makes broad suggestions for how animals might be appropriately incorporated in ethics guidelines.

## Introduction

Artificial intelligence (AI) ethics guidelines are intended to inform and influence the ethical design and use of this increasingly pervasive technology (Corrêa et al., 2023). These guidelines make strong and important appeals for the ethical treatment of human beings, who may be negatively as well as positively impacted by AI systems. For example, humans can be physically and emotionally endangered, discriminated against, and disrespected by AI uses and outputs (Eubanks, 2018; O’Neil, 2016). However, the same AI ethics guidelines largely overlook the vital interests of nonhuman animals that are sentient, by which we mean having a capacity for positive and negative subjective experiences such as pain, pleasure, and emotion. Sentient animals may be significantly affected by AI, for better or worse (Hagendorff, 2022; Mancini, 2024; Ryan, 2022; Singer & Tse, 2022). In this paper, we argue that AI^1^ ethics guidelines should not only inform and call for the protection and promotion of the interests of human beings in relation to AI use, they should also do something similar for sentient animals (Owe & Baum, 2021). For reasons we explain, it is time to rectify the omission of sentient animals and to properly include them in AI ethics guidelines.^2^

For the purposes of our argument, a precise definition of AI is not necessary. ‘AI’ is itself an umbrella term for a range of ‘techniques to enable software to approximate human thinking and behaviours’ (Yang et al., 2024) such as learning, reasoning, perceiving, problem solving, and creative synthesis. AI includes but is not limited to ‘machine learning’. AI also has various modalities like classification, recommendation, prediction, transcription and translation, decision-making, and text and image generation (Bender & Hanna, 2025, pp. 6–7; Narayanan & Kapoor, 2024). Many of these technologies may affect animals intentionally (e.g., AI systems that monitor and manage conditions inside confined animal farming operations), unintentionally (e.g., automated intelligent driving systems that seek to identify and avoid collisions with other road users), directly (e.g., automated systems for identifying and killing ‘pest’ species in wildlife areas or systems for communicating with animals), or indirectly (e.g., biases and speciesism coded into large language models) (see Coghlan & Parker, 2023).

What are AI ethics guidelines? These creations may encompass broad ethical statements, declarations, codes of practice, and collections of AI ethics principles (Corrêa et al., 2023; Jobin et al., 2019). They are often voluntary or non-binding, and may be produced by governments, intergovernmental bodies, supranational bodies, NGOs, academic groups, professional organisations, and for-profit corporations (Hagendorff, 2020; Prem, 2023; Schiff et al., 2020). In recent years, AI ethics guidelines have grown dramatically in number as concern about the nature and range of AIs ethical impacts has grown. Unfortunately, only a tiny number of these guidelines consider how AI might affect animals.

Like governments and intergovernmental bodies, some of the for-profit and civil society organisations that issue AI ethics guidelines are large and influential. The guidelines these bodies and organisations produce may be intended to influence research, design, governance, law, regulation, and use of AI across numerous domains (Ferrell & Ferrell, 2024). Although the effectiveness of AI ethics guidelines has been strongly criticised (Munn, 2022; Phan et al., 2022; Rességuier & Rodrigues, 2020), the promulgation of AI ethics guidelines has certainly generated much discussion about the ethics of the technology. It is at least arguable that the creation and implementation of AI guidelines might shape people’s moral understanding of AI’s effects and, over time, even sometimes lead to appropriate action (Larsson, 2020). Accordingly, it seems reasonable to ask whether AI ethics guidelines should say something about animals as well as humans.

We argue in this paper that AI ethics guidelines should explicitly and adequately refer to the moral value of individual sentient animals and the need to consider their interests when developing and using this powerful technology. Our case advances by anticipating the following five possible objections to including animals in AI ethics guidelines: 1) Animals are not significantly affected by AI; 2) Humans lack duties to protect or help animals; 3) Considering animals is overridden by more pressing duties to humans; 4) There is too much disagreement about how animals should be treated; and 5) Voluntary AI ethics guidelines are unhelpful or counterproductive. Such objections may be variously made by proponents and opponents of AI ethics guidelines. Our argument is that these five objections to sentient animal inclusion ultimately fail. Their failure strengthens the case for proper animal inclusion.

We begin with some background on AI ethics guidelines (Section “AI ethics guidelines”) and the absence of ethical reference to animal interests therein (Section “Guidelines and animals”). The argument then proceeds as follows. Like humans, animals may be variously harmed or benefitted by AI (Section “Objection one: ‘animals not significantly affected by AI’”). Sentient animals also have intrinsic, relational, and instrumental moral value (Section “Objection two: ‘no duties to protect or help animals’”). Notwithstanding significant moral duties to fellow human beings (Section “Objection three: ‘duties to animals overridden by duties to humans’”) plus societal disagreement about animals’ moral value (Section “Objection four: ‘too much disagreement about how animals should be treated’”), humans nonetheless have some responsibilities to protect animals from harm from AI and to try to ensure they receive some benefit from the technology. While voluntary ethics guidelines are certainly no panacea for steering and encouraging ethical AI, they might have some positive if modest effects (Section “Objection five: ‘AI ethics guidelines are unhelpful or counterproductive’”).

A key argument we make for greater inclusiveness beyond the species barrier is that animals are a profoundly morally and socially invisible group (Section “Objection five: ‘AI ethics guidelines are unhelpful or counterproductive’”). Animals’ comparative invisibility and marginalisation, we shall suggest, makes their incorporation in existing and future guidelines and discussion about AI important and urgent. Furthermore, leaving sentient animals out of AI ethics guidelines altogether, or making only glancing or obscure reference to these interest-holders, renders those guidelines less helpful overall than they could be. Towards the end of the paper, we offer some high-level suggestions about how to adequately bring sentient animals and their interests into AI ethics guidelines (Section “How animals might be included in AI ethics guidelines”), before summing up the argument (Section “Conclusion”).

## Background

Some background on AI ethics guidelines (Section “AI ethics guidelines”) and their omission of sentient animals and their interests (Section. “Guidelines and animals”) is necessary before we examine and counter objections against animal inclusion.

### AI Ethics Guidelines

There are now hundreds of AI ethics guidelines published by many and varied organisations. Although their production has recently slowed (Corrêa et al., 2023), these guidelines nonetheless remain prominent, widely available, and frequently discussed. Groups authoring AI ethics guidelines come from both the private and public sector, and include for-profit companies and tech giants, governments and their departments, intergovernmental bodies, supranational bodies, NGOs, professional computing and other organisations, think tanks, and academic groups (Hagendorff, 2020; Jobin et al., 2019; Prem, 2023; Schiff et al., 2020).

Some AI ethics guidelines focus specifically on obligations to particular groups such as children (International Research Center for AI Ethics and Governance, 2022) or medical patients (AHPRA, 2024). These more focussed guidelines cannot generally be expected to include animals. However, many AI ethics guidelines are intended to have a wider and more comprehensive application (Jobin et al., 2019). This is where the omission of animals and their interests becomes most salient.

AI ethics guidelines can manifest as ‘frameworks’, ‘statements’, ‘declarations’, and professional ‘codes of practice’ (e.g., the Association for Computing Machinery’s or ACMs code).^3^ These documents tend to provide reasonably high-level and general^4^ ethical advice on the design, development, deployment, and running of AI systems. By advancing broad ethical norms, values, or principles, such guidelines aim to cover many possible forms of AI and circumstances of AI use. Examples of commonly encountered AI ethical principles include safety, fairness, privacy, transparency, accountability, and non-maleficence (Hagendorff, 2020; Jobin et al., 2019).

Most AI ethics guidelines are set out as statements of values or principles with no binding power of their own. To have full effect, they must be observed and implemented, which will often depend on the power and influence of the authors or sponsoring organisation. The ways in which such codes or guidelines may be implemented include being purely educational or discursive and aimed at indicating a voluntary commitment, making demands of other organisations, guiding internal processes and procedures, being formalised as disciplinary rules with sanctions for non-compliance within an organisation, profession, or industry, and being formally recognised by the law as good practice or as sufficient evidence of compliance with regulatory requirements (Gießler & Hass, 2020).

Proponents of AI ethics guidelines may believe that statements of values or principles, even if not backed by ‘hard’ legal force, can have practical value. Proponents may believe, for example, that guidelines could instigate a conversation within an organisation, industry, or profession, or in society more generally. Such conversations and dialogue could cover various growing risks posed by AI systems, how decision-makers and interest-holders can and should address these risks, and how to better promote the benefits of AI for individuals and society.

As we noted, most AI ethics guidelines today are advisory and voluntary only, and many provide general ethical recommendations that apply outside the power of the issuing organisation to enforce. Nonetheless, some guidelines, such as those from professional organisations and governments, may also have additional regulatory force. For example, health and legal professional organisations are now beginning to publish principles for how to use and not use AI systems as part of their professional codes, which can be taken into account in disciplinary proceedings (ABA, 2024; AHPRA, 2024). And governments have promulgated aspirational codes or guidance which may later be hardened into guiding values for legislation or be recognised as best practice in regulatory guidelines (Griffin, 2024).

Furthermore, individual companies have the option to internally enforce compliance with their declared values. Given the rapid innovation and growth of AI itself, AI policy and regulation is in flux. The promulgation of AI ethics principles and values by powerful companies and governments and by industry, professional, and civil society groups could arguably have a significant influence on the shape and nature of future laws, policies, and governance institutions as they rapidly develop (Brownsword, 2022).

AI ethics guidelines, however, have their critics. Some critics argue that such moral statements and principles, at least when they are voluntary or not legally binding, have no worth or have at best a dubious practical value (Corrêa et al., 2022; Kazim & Koshiyama, 2021; Mittelstadt, 2019; Munn, 2022). An even more damning criticism is that ethical declarations and guidelines of these kinds are positively counterproductive to the goal of ensuring genuinely safe and ethical AI (Héder, 2020). We shall later address these critiques in the context of inclusion of sentient animals.

### Guidelines and Animals

Despite often attempting to be general and comprehensive, AI ethics guidelines rarely mention sentient animals as possible ethical subjects of AI’s impact. Many guidelines are explicitly and decidedly anthropocentric. Often, this human-centric orientation rightly recognises AI risks against a backdrop of commercial, national, and technological imperatives to innovate and capitalise on AI (Seger, 2022). In this competitive and frequently fast-paced environment, talk of ‘human-centered’ AI reminds people that technological development should serve humans and society, not just the technology’s owners. But this human-centred orientation can also mean that other animal species are passed over.

In fact, nonhuman animals have been mostly missing in AI discourse more generally (Singer & Tse, 2022). Some now argue that AI technical and ethics communities have largely overlooked the morally relevant effects that the technology can have on sentient nonhuman animals in farming, science, vehicle automation, and many other circumstances (Coghlan & Parker, 2023; Hagendorff, 2021; Nandutu et al., 2023; Owe & Baum, 2021; Simoneau-Gilbert & Birch, 2024). These scholars claim that sentient animals are owed some duties and should be considered in the development of AI and/or related technologies (such as automated robotics) that also affect animals (see Sparrow & Howard, 2021).

Previous research (Rigley et al., 2023; Singer & Tse, 2022) has shown that most AI ethics guidelines omit animals and their interests. Consider some examples. AI ethics guidelines emerging from some prominent intergovernmental bodies, such as the Group of 20 (G20),^5^ make no explicit reference to animals and AI’s possible impact on them. Ethics guidelines from large tech companies, such as Microsoft^6^ and Google,^7^ also typically omit reference to the importance of protecting or benefiting animals with respect to AI technologies.

While some guidelines acknowledge AI’s impact on the *non*human world, they may do so in terms of the ‘environment’ rather than in terms of sentient animals as individual ethical subjects. A government-level example is the Australian government’s *AI Ethics Principles,* and associated *Voluntary AI Safety Standard,* which both refer to ‘human, societal and environmental wellbeing’ without mentioning animals.^8^ At the International level, the OECDs AI Principles include ‘sustainable development and well-being … for people and planet’.^9^ But sentient animals as individuals who may be harmed or benefitted by AI are left out.

Talk of ‘sustainability’ and ‘environmental wellbeing’ in these guidelines could *potentially* include animals, but it is also compatible with ignoring animal interests. Typically, discussion of environmental and sustainability issues refers to the energy and water usage of data centres supporting AI and the potential for AI-enabled critical infrastructure to malfunction and harm the surrounding environment. It also sometimes refers to matters of material extraction and end-of-life waste in the infrastructure and equipment needed for AI development and deployment (Cellard et al., 2025). Although these factors are certainly of indirect significance to animals, direct ethical consideration of sentient animals is frequently absent in guidelines.

When animals are mentioned at all, this may be done in relatively minor or glancing ways. For example, UNESCO’s four core values in its *Recommendation on the Ethics of Artificial Intelligence* include as the fourth value, ‘environment and ecosystem flourishing’.^10^ While not featured on the public facing webpage,^11^ animal welfare is cited as being potentially impacted by AI technologies in the preamble of the full text.^12^ The supporting policy recommendations concerning ‘environment and ecosystem flourishing’ point out that ‘environment and ecosystems are the existential necessity for humanity and other living beings to be able to enjoy the benefits of advances in AI’.^13^

Similarly, the Institute of Electrical and Electronics Engineers or IEEE, the world’s largest technical professional organization, refers to animals in the introductory material for its 250-odd page guidelines, ‘Ethically Aligned Design: A Vision for Prioritizing Human Well-being with Autonomous and Intelligent Systems’. This document speaks of the ‘consequences’ of intelligent machine action ‘for humans, for animals, for things in the environment, and, finally, for the machine itself’.^14^ However, animals are not otherwise mentioned or discussed in the document’s list of ethics principles.

Some guidelines have given a little more prominence to animal interests by including them in their core or general ethical principles. For example, the European Commission’s 2019 ‘Ethics Guidelines for Trustworthy AI’ asks AI developers or deployers to ‘consider the potential impact or safety risk to the environment or to animals’.^15^ It additionally states that ‘[p]reventing harm also entails consideration of the natural environment and all living beings’.^16^ These Guidelines were an important precursor to the European Union’s groundbreaking *Artificial Intelligence Act* (2024) (‘EU AI Act’).^17^ However, while environmental protection was included as an objective of the EU AI Act,^18^ animals were not mentioned in the Act. Animals have subsequently been very briefly, and somewhat tacitly, included in the voluntary *Code of Practice for General-Purpose AI Models* (2025), created by a multi-stakeholder expert group under the aegis of the European Commission to help industry comply with their obligations under the EU AI Act. As a result of representations that the relevant risks should include risks to animals from AI, that Code refers to risks to ‘non-human welfare’ as an example of a type of systemic risk that industry should identify as part of their risk assessment obligations^19^.

The ‘Montréal Declaration on Responsible AI’, developed in 2017 after the Université de Montréal conducted citizen deliberations, also refers to animals in its key ethics principles. The first of the Declaration’s 10 core ethical principles, the Well-being Principle, declares that the ‘development and use of artificial intelligence systems (AIS) must permit the growth of the well-being of all sentient beings’.^20^ The explanation of the principle also says that AI ‘must allow individuals to pursue their preferences, so long as they do not cause harm to other sentient beings’.^21^ Again, this certainly gives animals some prominence in comparative terms, especially since animals or ‘sentient beings’ are not just mentioned but also included in a core ethical principle. Nevertheless, such examples are exceptions to the norm. We will now argue that animals should be both included in AI ethics guidelines and given adequate recognition within them.

## Animals in AI Ethics Guidelines: Addressing objections

The suggestion that sentient animals should be adequately recognised in AI ethics guidelines may not be obvious to many, and some technology companies and other organisations who develop AI guidelines may not be convinced. To build our case for such recognition, then, we now anticipate and evaluate five key objections to animal inclusion.

### Objection One: ‘Animals Not Significantly Affected by AI’

Classic AI controversies such as racially biased facial recognition, unjust allocation of welfare payments, unsafe social media algorithms, and discriminatory policing recommendations (Eubanks, 2018; O’Neil, 2016) may suggest that AI is a purely anthropocentric or a human-only affair. Furthermore, general AI discourse has devoted little space to discussing animals as legitimate interest-holders who can be affected by machine-learning and intelligent automation. However, several scholars have shown that animals can and will be significantly impacted by AI (Hagendorff, 2021; Owe & Baum, 2021; Singer & Tse, 2022).

Such scholars note that impacts of AI on sentient animals can involve both harms and benefits to their interests (Bossert, 2023; Coghlan & Parker, 2024). The term ‘interests’ here refers to what makes a sentient animal’s life go well or badly (see also section “Objection two: ‘no duties to protect or help animals’”). Various thinkers have recognized that animals have interests in experiencing positive feelings and emotions, avoiding aversive experiences and suffering, engaging in desired activities, pursuing relationships with their own or other species including humans, maintaining group practices and nonhuman cultures, and not losing their lives pre-maturely (Donaldson & Kymlicka, 2011; Gruen, 2014b; Nussbaum, 2023; Regan, 2004; Singer, 2023). AI could potentially damage or promote all these interests—AI thus has a morally ‘double-edged’ nature or potential. Consider some examples.

AI may be used to avoid harming animals or making them suffer by replacing animal models of physiology or disease. That shift could prevent some of the harming and killing of millions of animals that routinely occurs in science (Mancini, 2017). AI will also likely improve veterinary care (Coghlan & Quinn, 2023) by enabling more accurate testing, imaging, monitoring, and diagnosis of animal patients (Burti et al., 2024). However, AI might also harm animals in medical and veterinary research and science more generally. An example reported in the media was the suffering and death of many animals in the development of Neuralink’s brain-computer interface (Ryan, 2022), a project in which devices were implanted into the brains of pigs and monkeys to translate neural signals into actions (R. Levy, 2022).

AI may also both harm and benefit animals in agriculture, most conspicuously through the intelligent monitoring and automation of farming activities (Neethirajan, 2021; M. Ryan, 2019; Ziesche, 2021). For example, AI may be used to automate activities like feeding animals according to machine-based inferences about their specific needs—for example, by computing data such as their feeding frequency and bodyweight (Jia, 2022; Mahfuz et al., 2022; Wurtz et al., 2019). No previous technology could make such precise inferences and automate the response without needing a ‘human-in-the-loop’. AI might also support round-the-clock monitoring of signs of disease signs like coughing and lameness and even affective states amongst large numbers of animals (Antanaitis et al., 2020; Džermeikaitė et al., 2023; Karthick et al., 2020; Singh et al., 2022)—something that farmers could not feasibly accomplish without such technology. Such AI might thus identify problems that could otherwise be missed. This may improve animal welfare, although only when such information is appropriately acted on or automated.

However, AI inaccuracy and machine bias in any of these activities may damage animal welfare (Sparrow et al., 2021). Moreover, commercial imperatives could lead to AI being marshalled to intensify living conditions for animals, such as through enabling stricter animal confinement and facilitating the growth of more intensive factory-style farms, including multi-storey indoor facilities with many times more animals (Staff, 2022; Simoneau-Gilbert & Birch, 2024). Increasing AI automation in farming operations could also arguably have more indirect effects. For example, if animal production systems are largely run by smart machines rather than human beings (farmers) who care for the animals by interacting with them, it might have the effect of further objectifying animals and alienating us from them (Bossert & Coeckelbergh, 2024).

Alienation could occur at two levels. First, farms in which people care for and interact with their animals may be replaced by more impersonal automated farms, causing alienation at a local level. Second, if fully automated farms become the norm, the public may lose a sense of farmed animals as beings which people care for and about. That could mean a much broader level of moral alienation and objectification, which could act to reverse the growing public interest in farmed animal welfare (Sinclair et al., 2022).

Wild animals too may be affected for good and ill by this technology. For example, AI can be recruited to more accurately identify wild or free-living animals to protect them (Nandutu et al., 2023). Yet this technology can also be used to augment poaching, hunting, and harassment of wild species. AI is being used to target ‘invasive’ species and deliver lethal baits in intelligent traps that identify an animal’s species (Charlton et al., 2023). But in addition to causing often extreme suffering to targeted animals, inaccurate machine recognition of animals could lead to the poisoning and associated suffering of individuals from non-targeted and protected species (Rickards et al., 2022).

There are other examples of AIs double-edged potential. AI might, for instance, assist understanding of the nuances of animal languages, such as whale, dolphin, and bat echo-communication. Such understanding could allow us to better protect these animals (Ryan & Bossert, 2024). But at the same time, this techno-communicative advance may also allow humans to more readily interfere in their lives to the animals’ detriment.

Large language models (LLMs) could permit people to learn more about animals and their interests. Once again, however, this development may be two-edged, since LLMs might also produce biased and ‘speciesist’ outputs (Ghose et al., 2024; Hagendorff et al., 2022; Luccioni & Rolnick, 2023). An example of this bias is when LLMs portray some sentient animals as killable, disgusting, worthless, or mere pests. Though machine-generated words may seem innocuous, such language may perpetuate negative attitudes towards animals that lead to them being harmed or neglected over longer time scales. While negative portrayals of animals already occur, popular LLMs and chatbots may spread these depictions quickly and on a massive scale. Finally, social media algorithms could stimulate and amplify the production of graphic video content involving animal exploitation, cruelty, and abuse Coghlan and Parker (2023)).

AI is already significantly affecting animal interests, often indirectly via effects on human behaviour, for better and for worse. Its impacts on animals may grow alongside likely increases in the technology’s power and reach. We agree with other scholars that the positive and negative potential of AI for sentient animals requires some moral thought and accounting.

### Objection Two: ‘No Duties to Protect or Help Animals’

However, some may doubt we have moral duties to protect sentient nonhumans from AIs impacts and that only humans warrant protection. Accordingly, it would be unnecessary to include animals in AI ethics guidelines. We shall present three basic ethical reasons for recognising duties to protect and assist some animals on some occasions. These reasons relate to intrinsic moral value, relational features, and instrumental moral value. Such reasons have, of course, been extensively defended (Armstrong & Botzler, 2017; Donovan & Adams, 2007; Singer, 2006). Although these reasons will be familiar to philosophers, we briefly present them here to counter the current objection to animal inclusion.

Intrinsic moral value implies that animals have ethical value in their own right, not merely value for other purposes (Regan, 2004). Animals are ‘morally considerable’ or have ‘moral status’ and warrant moral attention for their own sakes (Jaworska & Tannenbaum, 2021). Animals may be *wronged* when treated in certain ways. In contrast, it is surely impossible to wrong, say, a computer by smashing it. Computers lack intrinsic moral value. Of course, smashing a computer may wrong its human owner, but mistreating an animal does not merely wrong humans associated with the animal. Rather, harmful treatment can wrong the animal itself. Similarly, we may sometimes have responsibilities to *help* an animal for their own sake by, for example, getting them veterinary assistance.

A widely accepted basis for recognising the intrinsic moral value of animals relates to *sentience* (DeGrazia, 1996). Possessing sentience, many argue, is a sufficient condition of being intrinsically valuable.^22^ Mammals, birds, reptiles, and perhaps other animal groups are thought to be sentient (Browning & Birch, 2022). Certain animals can, for example, feel pain, fear, distress, pleasure, and contentment. These sorts of experiences are often considered to be part of an animal’s *welfare*. Objects like computers are not sentient and cannot qualify on this ground as having intrinsic moral value^23^ or a welfare.

Some people think that sentient animals typically also have interests in the form of preferences. For example, animals may prefer to avoid aversive experiences, engage in certain activities like play, and maintain relationships with others (Mellor, 2017; Nussbaum, 2006). Such preferences may also connect to an animal’s welfare (Singer, 2023).^24^ An animal with sentience and preferences can be wrongly or rightly treated when their welfare interests are affected in certain ways. To be clear, recognising intrinsic value does not imply we may *never*, for example, harm animals. Sometimes harming can be morally justified. Nevertheless, damaging an animal’s welfare always requires a good moral reason.

Some critics argue that sentience is insufficient for possessing intrinsic moral value because that value requires higher capacities such as rationality or self-consciousness (Kant & Wood, 2002; Leahy, 2005). Most philosophers, however, would disagree (Gruen & Monsó, 2024). Consider two reasons. First, one could appeal to the basic moral intuition that experiences like suffering matter ethically. Our moral responses towards, say, torturing a dog and smashing a computer seem very different. Philosopher Christine Korsgaard (1996, p. 153) suggests we ‘no more hear the cries of an animal as mere noise than [we do] the words of a person’. This response to suffering does not require that the sufferer is, say, fully rational (Bentham et al., 1996). Second, philosophers argue that some humans, such as people with severe mentally impairments, also lack rationality (Pluhar, 1987), yet surely do not lack intrinsic moral value. These replies put some argumentative onus back on the critics.

The second important ethical reason for protecting and benefitting animals concerns morally relevant *relations* (May, 2014). The idea that sentient animals have intrinsic moral value implies that we can have direct ethical duties to animals with whom we have no special relationship, such as free-living animals in wild and urban environments. However, we may have more numerous and stringent duties to animals with whom we have certain relations (Palmer, 2010). For example, some animals are our companions or depend on humans for their existence and survival (Serpell, 1996; Williams et al., 2024). We thus have some special relational duties to animals in farming, science, entertainment, and households (Donaldson & Kymlicka, 2011).

The third moral reason for attending to animal interests concerns instrumental value. Unlike intrinsic moral value, which relates to duties to animals for their own sakes, instrumental value is connected to duties to protect or promote animal interests for the sake of something else. That ‘something else’ could, for example, be biodiversity, the environment, or humans themselves (Brennan & Lo, 2002).

In fact, human and animal welfare are often interconnected (Pinillos et al., 2016; Zinsstag et al., 2011). For example, our mental health may be aided by interaction with wild and domesticated animals (Curtin, 2009). Another example of human-animal interconnectedness relates to disease and the environment. Certain animal agriculture systems and associated land clearing not only harm billions of animals but simultaneously heighten risks of novel zoonotic pandemics (Centers for Disease Control and Prevention, 2022; Dhont et al., 2021) and climate change (Eisen & Brown, 2022). Conversely, reducing industrial animal agriculture that harms animals and protecting wild species from death and injury may reduce harm to humans from disease, carbon emissions, and ecosystem collapse (Gibb et al., 2020; Napolitano Ferreira et al., 2021). Such connectedness creates various instrumental duties to animals beyond our direct ethical duties to them.

In sum, intrinsic, relational, and instrumental value are three widely discussed moral reasons for taking AI’s impacts on animals seriously. Since AI may both harm and benefit sentient animals, AI developers, deployers, users, and regulators have some duties to protect and even benefit sentient animals when they produce, operate, endorse, or permit the development of such technology (Tasioulas, 2022).

### Objection Three: ‘Duties to Animals Overridden by Duties to Humans’

Now, however, another possible objection emerges. Even if it is granted that sentient animals possess intrinsic moral value, some critics will nonetheless argue that our duties to protect animal interests and welfare are extremely rare duties at best. Animal-related duties, these critics might say, are almost always outweighed or silenced by other moral duties, principally duties to human beings. Accordingly, we may, and perhaps should, largely ignore animal welfare or interests in practice. Philosopher Mary Midgley (1983) characterises this attitude as the *relative dismissal* of animals’ moral claims. Whereas *absolute* dismissers regard animals as not being morally considerable at all, relative dismissers think that while animals have some intrinsic value, we can and even should mostly ignore their interests in practice.

One reason behind relative dismissal is the belief that the intrinsic moral value of animals is real but marginal or slight. A proponent of this view might claim that the moral worth of sentient animals is like that of, say, a jellyfish, which is (let us assume) non-sentient. Let us suppose, this person might say, that a jellyfish or some other simple living thing has, despite its lack of sentience, a non-zero intrinsic moral value but a value that is nevertheless still very low. While it may be wrong to injure or kill insentient animals with non-zero but low moral value for fun, this, the critic may claim, is a relatively weak duty, easily overridden by other duties.

However, philosophers who hold that sentient animals have moral value do not usually believe that this value is marginal or slight as it arguably might be for insentient living things like jellyfish. Instead, they tend to think that sentient animals have a more substantial or weighty ‘moral significance’ (Goodpaster, 1978). This does not mean that sentient animals have the same moral significance as humans—for humans and animals may not have equal moral value—but it does mean we should take sentient animal interests and welfare seriously (DeGrazia, 1996).

Such a position might be defended in a variety of ways, including by again appealing to our basic intuitions (Pust, 2024). For example, our considered view may be that killing or badly injuring a dog or monkey is significantly more wrong than badly injuring a jellyfish. We may agree that we need significantly weightier moral reasons—certainly reasons well beyond, for example, sheer convenience—to justify harming dogs compared to harming animals like jellyfish that are (arguably) insentient.

Another possible reason for dismissing in practice the needs and interests of animals is that it prevents or deflects us from fulfilling even stronger duties to humans. Midgley (1983, p. 31) believed that such relative dismissal can be driven by the assumption that human compassion is easily exhausted like a rare precious fluid. Compassion must therefore be tightly rationed. Midgley considers this view a myth. Indeed, animal-oriented compassion might accompany or even augment human-oriented compassion. Early defenders of animals, for example, also campaigned vigorously for children’s and women’s rights.

A related objection is that devoting time and money to protecting animals wrongly reduces these resources for promoting human wellbeing. But this particular human-centred stance also has problems. First, there are many animals experiencing not just small harms but severe suffering and deprivation, including billions of sentient animals in restrictive confinement systems (Fraser, 2012; Williams et al., 2024). Their needs surely have some moral urgency too. Second, protecting animals from various sources of harm, including harm caused by AI, does not always conflict with human wellbeing. For example, various anti-cruelty laws do not necessarily impose heavy burdens on humans.

Third, as we observed earlier when discussing instrumental duties, human and animal interests often coincide and connect. For example, some kinds of animal agriculture promote zoonotic corona and flu viruses (Hayek, 2022) and accelerate dangerous climate change (Steinfeld et al., 2006). AI might help here by, for example, accelerating the development of plant-based meat substitutes and other animal product alternatives (St. Pierre et al., 2024). Some recent research also suggests that a domination mentality over animals is linked to human prejudices, such as racism (Dhont et al., 2016). AI could conceivably be involved in promoting, or else limiting, such attitudes. For example, generative AI could foster, or else oppose, supremacy thinking (Kymlicka, 2018) toward animals that arguably helps sustain human-oriented bigotries. Furthermore, replacing harmful animal studies which extrapolate poorly to human beings with powerful AI simulations of human bodily systems, might benefit humans and animals together (Gangwal & Lavecchia, 2025).

### Objection Four: ‘Too Much Disagreement About How Animals Should be Treated’

Despite the above arguments, it is certainly true that moral disagreement exists in society about the extent to which we should protect animal interests and lives. As we saw, people differ about the strength of our direct moral duties to animals. Some people are ‘relative dismissers’ of animals and their interests (N. Taylor, 1999), while others recognise more significant animal-directed duties (Midgley, 1983). Still others believe animals and humans are owed equal moral consideration (Singer, 2023). By contrast, while there is certainly ethical disagreement over some ethical duties to humans (Mittelstadt, 2019; Ryan & Stahl, 2021), it is much more limited than it is for animals.

Disagreement about the nature and strength of our duties to animals can result in major differences in attitudes about when and how we should protect them. For example, some people, but not others, will tend to think that in general only illegal forms of treatment of animals are morally wrong, and that all or most currently legal uses of animals, however harmful to them, are morally acceptable and should be allowed. Some people, but not others, will think it acceptable to use AI to make intensive farming still more intensive, even though the animals will suffer more seriously (Leahy, 2005). Some people, but not others, will think it morally permissible to use AI to poison millions of animals in conservation (Coghlan & Cardilini, 2022). And so on.

Given this diversity of viewpoints, it might be argued that we should refrain from instructing or even simply advising others about how they should treat sentient animals. This conclusion could be said to follow from the need to show democratic and cultural respect for divergent beliefs and values. Because of the need for such respect, a critic of our position may say, AI guidelines should stay largely neutral and silent about how AI ought to be used when it comes to its possible impacts on animals.

This objection may, however, be questioned. For a start, moral truth surely does not depend simply on agreement or consensus amongst people within societies or cultures: most of us would think that a moral position that is disputed or controversial or is a minority position can still be correct and that moral truth is not merely ‘relative’. Furthermore, although showing various forms of respect for the moral beliefs of other individuals or cultures is ethically important, it does not uniformly require moral neutrality or silence, including when individuals with intrinsic value stand to be victims of serious harms or to miss out on significant benefits which they are owed. We recognise this, of course, in the human case. The same logic applies to animals to whom we owe significant moral duties, even if, once again, it is not the case that humans and animals are moral equals.

It is also worth saying that despite moral disagreement about sentient animals, societal support for protecting animals is growing (Sinclair et al., 2022), even though the public is often unaware of many details of animal treatment and the extent to which many animals suffer (Cornish et al., 2016). The general population may therefore have mostly positive attitudes towards the inclusion of animals in AI ethics guidelines. In turn, such guidelines might help to remind or inform us about the ways in which animals are at risk, including from AI technologies. Furthermore, recognising animals in this way might also benefit an organisation’s reputation and social license (Morrison, 2014).

That said, comparatively higher levels of disagreement about duties to nonhuman animals may rightly affect the details of how AI ethics guidelines are crafted. That is because such guidelines are aimed not just at reflecting moral truth or stating important ethical duties but also at having some influence on others’ beliefs and behaviour. Thus, if a more conservative stance on certain kinds of animal protection will likely garner greater support or buy-in than a more progressive stance, then, even if the stronger stance is the right one, the more moderate stance may be chosen for strategic purposes.

The nature of the organisation issuing ethics guidelines may also affect what types of animal protection is appropriate or strategically wise to include. For example, biomedical and pharmaceutical scientists and their professional organisations might focus on ethical obligations to use AI to replace and reduce impacts on animals used in experiments, and organisations producing AI systems for agricultural applications could specifically develop animal welfare guidelines for farming. Organisations that do not have specific entanglements with animals could take a more general approach.

### Objection Five: ‘AI Ethics Guidelines are Unhelpful or Counterproductive’

So far, we have argued that animals can be harmed and benefited by AI (and other technologies) and that there are corresponding and real ethical duties to protect and advance sentient animal interests. The next objection, however, can acknowledge that all this is true yet still claim that there is no need to include animals in AI ethics guidelines. The argument for this position is that AI ethics guidelines are simply morally unhelpful—or worse, even counterproductive. If that is so, then perhaps it even follows that the more we think that animals morally matter, the less they should be included in these guidelines. Whereas the previous four objections might well come from authors or proponents of AI ethics guidelines, this fifth objection comes from *general critics* of AI ethics guidelines.

Ethics guidelines have been heavily critiqued for failing to protect vulnerable *humans* from AIs pernicious effects (Munn, 2022). An essential problem, critics say, is that these guidelines are merely voluntary or ‘soft’ in nature. Critics have thus attacked guidelines for lacking genuine teeth. Furthermore, these opponents argue, ‘soft’ value statements enable the mere pretence of ethical behaviour. The upshot is that various organisations’ guidelines are often merely ‘high-minded’ virtue signalling (Rees & Müller, 2023) or moral grandstanding (Tosi & Warmke, 2016). Worse still, critics see them as being distracting and as derailing more important and effective approaches to protecting people from technology (Hass & Gießler, 2020).^25^

Critics thus allege that merely voluntary or aspirational guidelines can be dangerous exercises in ethics washing (see Bietti, 2021). As such, guidelines can prevent necessary changes in the law to truly safeguard human interests. Strong regulation, critics maintain, is required to achieve adequate protection from AI’s many human harms. Yet deceptive behaviour can stand in the way of these necessary legal changes. Such deception or obfuscation, opponents tell us, is facilitated by AI ethics guidelines and especially benefits tech corporations and other businesses (Kayser-Bril, 2019; Phan et al., 2022).

This human-centred criticism of AI ethics guidelines logically extends to our case for including animals. In other words, the criticism here could be that profit-oriented businesses might use ethics statements to cynically avoid scrutiny and deter harder regulation of AI that is harmful not just to humans but also to animals. Of course, not all organisations are insincere or self-serving. Leaders and members of many professional, academic, and intergovernmental organisations may be genuine about their stated values. Still, it is true that ethics washing and deflection concerning animals and AI is possible, especially when a government or a corporation is committed to rapidly progressing AI products and industries. To justify animals’ recognition in various AI ethics guidelines, we need an effective countervailing reason to outweigh this reasonable concern.

In our view, there is such a reason, and it is key to our argument for animal inclusion. This reason is related to the fact that sentient animals are often overlooked by human beings to a remarkable degree. Mary Midgley (1983) also wrote about this issue. Midgley believed that the complete or ‘absolute dismissal’ of animals (as opposed to ‘relative dismissal’) has reduced in contemporary times. Views that totally disregard animals have been attacked, at least in some places. For example, Descartes’ view that animals are mere insentient automata, and Spinoza’s view that concern for animals is ‘womanish pity’ (Midgley, 1983, p. 10), have been repeatedly challenged in science and philosophy (Birch et al., 2020; Singer, 2023)

Nonetheless, many people around the world are still either absolute or relative dismissers of animals. Despite increasing concern in many places for animal welfare, in practice it remains all too easy to ignore or forget animals, especially when so many fellow humans need our assistance (Midgley, 1983). Another philosopher, Carol Adams (2000), has used the term ‘absent referent’ to highlight the way animal interests are often forgotten even when animals’ presence should be obvious. For Adams, this occurs most glaringly in our discussions about preparing and eating meat. A key point made by these philosophers is that, notwithstanding significant moral progress in attitudes to animals in recent decades, animals often remain a morally and socially invisible group of interest-holders (Tovey, 2003; Vinnari & Vinnari, 2022).

The invisibility of animals shows tellingly in their very absence from AI ethics guidelines and discourse (Singer & Tse, 2022). This often-unnoticed omission from guidelines not only reflects but also arguably perpetuates the relative moral facelessness of animals. Because even today we only occasionally see animals presented in moral terms, and because animals are so often missing from our perceptions, discussions, and activities, the chances are increased that we will continue to sideline them and allow them to be wrongly harmed.

The need to address and reverse the current invisibility and marginalisation of animals is a compelling reason for recognising animals in AI ethics guidelines, despite genuine worries about ethics washing. Even more important than avoiding such deception is for animals to be publicly considered rather than pushed further into the outer shadows of moral concern (Midgley, 1983). Indeed, repeated ethical references to animals across various AI guidelines may serve as useful moral reminders for tech developers, regulators, and others.

It seems fair to say that frequent reference to nonmaleficence, safety, justice, transparency, and other norms have raised the profile of these AI ethics principles. Promulgation of these principles in guidelines has helped shape AI discourse, and it might even have some influence, however modest, on AI development (Gießler & Hass, 2020). Certainly, it is important not to overstate the practical influence and impact of AI ethics guidelines. As an example, one research study showed that ethics guidelines did not immediately influence a group of engineering practitioners and students (McNamara et al., 2018) who answered ethical questions about technology soon after they were exposed to that ethical guidance (but cf. den Bergh & Deschoolmeester, 2010; Schiff et al., 2020).

Nevertheless, guidelines might have more indirect effects by promoting awareness and conversation. Words sometimes have material consequences (Austin, 1975). When public espousal of moral values appears sincere, it may help foster agreement and a more ethically desirable culture in organisations and the community (Lauer, 2021; N. Levy, 2021; Seger, 2022). Indeed, it is possible to underestimate as well as to overestimate ethics guidelines’ influence (Gießler & Hass, 2020). In practical terms, ethics guidelines are an important way to foster discussion about AI and animals.

We want to again emphasise that moral awareness-raising is particularly important for marginalised and invisible groups. We already know this, of course, from former and current movements for racial and gender equality, for greater recognition of individuals living with disabilities, and for justice for peoples in the Global South or Majority World (Nussbaum, 2006)—all groups which, incidentally, may be inequitably impacted by AI (Eubanks, 2018; Png, 2022). In time, animal-related commitments from numerous and influential organisations might just help foster a wider view that animal interests should be considered when powerful technology is developed and implemented.

Sometimes, an organisation’s ethical declarations might also be used to promote accountability more directly. For example, employees of Google successfully (at least initially) protested the company’s involvement in the US defence department’s Project Maven—which was developing AI for military purposes (Crofts & van Rijswijk, 2020)—by referencing its public pledges not to ‘be evil’ or to not develop ‘[w]eapons or other technologies whose principal purpose or implementation is to cause or directly facilitate injury to people’.^26^ Analogously, if a company that was publicly committed to recognising animal welfare began creating inhumane AI systems, employees and other parties may remonstrate the contradiction, and could even be able to sue for misleading conduct if the company had represented itself in commercial contexts as protecting animal welfare—as for example, a company selling animal monitoring equipment might do.

In the long run, we must acknowledge, voluntary corporate led AI ethical guidelines are unlikely to be anything near sufficiently effective at ensuring AI benefits and does not harm animals on their own. While AI related companies, industry associations, and professional associations certainly should voluntarily adopt and systematically implement ethical guidelines that explicitly morally value animals, many, perhaps the majority, will likely only take their responsibilities to animals seriously if they are incorporated into government mandated and legally enforceable regulatory standards for which they can be held externally accountable (Norman, 2011; Parker, 2002).

Importantly, however, legally enforceable regulations often draw on pre-existing ethical codes and voluntary self-regulation for their content. For example, the European Commission’s ‘High Level Panel of Experts AI Ethics Guidelines’ informed the negotiation of the European Union’s landmark *Artificial Intelligence Act* (‘EU AI Act’) and supported the (belated) inclusion of ‘environmental protection’ as a guiding principle for that law (Hacker, 2024). They were also used to support the inclusion of ‘non-human [animal] welfare’ as an area for risk assessment in the *Code of Practice for General-Purpose AI Models* under that Act, as we mentioned above. This suggests that it is worth advocating for the inclusion of animals in prominent ethics guidelines insofar as these can also be used to scaffold and support their later inclusion in mandatory regulation.

It is well established that business corporations typically seek to create self-regulatory codes or ethical guidelines to stave off harsher regulation in the future, or to influence the terms of impending legal regulation (Bromberg, 2021; Ochigame, 2022; Phan et al., 2022; Yadin, 2025). This is a reason to be sceptical about the effectiveness and legitimacy of voluntary and internal guidelines created by for-profit businesses and industry associations. It is also simultaneously a reason for animal advocates and those without vested interests to strategically engage with—and as necessary to vigorously contest or propose animal-inclusive alternatives to—business and industry guidelines. Finally, it is possible to discuss the desired content of voluntary ethics guidelines and also advocate for external accountability and legally enforceable regulation at the same time (Rességuier & Rodrigues, 2020).

## How Animals might be included in AI Ethics Guidelines

We have now made the case for animal inclusion. This brings us to the question of how that might best be done. Including sentient animals in AI ethics guidelines across companies, governments, and civil organisations could be undertaken in various ways. At a broad level, tech companies might commit to not developing AI for purposes that unnecessarily and unjustifiably harm animals, and professional organisations might advise their members accordingly. Such entities could also indicate the desirability of investing in and exploring AI that alleviates existing animal harm and promotes better lives for animals. Academic, intergovernmental, and other bodies could commend ethical values of animal protection and benefit to AI companies, designers, deployers, and regulators.

However, the specific ways in which animals are spoken of and included in guidelines could matter. Ill-judged, unclear, vague, or manifestly unserious mentions could undermine or miss an opportunity to protect animals (Danks, 2022). Of course, each organisation must consider their own circumstances in framing ethics documents. As we noted, an organisation’s specific values and activities may guide their precise position. For example, an organisation committed to environmental goals might naturally take a strong stance against AI that treats animals in morally dubious ways that also damages ecosystems and climate. An animal advocacy or animal welfare organisation might take a specific interest in relation to the use of AI to expand animal agriculture industries or to further confine animals and restrict them from their ability to engage in natural behaviours (Bos et al., 2018; Tuyttens et al., 2022; Werkheiser, 2018).

A suitable framing of animal interests in AI ethics guidelines would ideally avoid fleeting and easily overlooked references to animals. The space devoted to animals and the tone taken in talking about them should display ‘signs of seriousness’ (Schiff et al., 2020, p. 158). Guidelines could point out that protecting animals sometimes (if often unexpectedly) also protects important human and environmental interests (Crary & Gruen, 2022; Lederman, 2024). But it will also acknowledge in some serious fashion that many animals are sentient, capable of feeling pleasure, pain, and emotion, and that sentient animals have intrinsic moral value and should be respected for their own sakes. In fact, acknowledgement of animal sentience and associated ethical requirements have been regarded as a good starting point for animal protection (Kotzmann, 2023).

Earlier, we quoted the Montréal Declaration’s Well-being principle that AI ‘must permit the growth of the well-being of all sentient beings’. We also explained the widely accepted view amongst philosophers (and others) that sentience matters in an ethical sense. Sentient animals have morally important interests in avoiding suffering, deprivation, and premature death (Nussbaum, 2023). Some AI ethics guideline authors may additionally choose to say that animals warrant human compassion and even justice (Nussbaum, 2006) and require respect for their dignity (Coghlan, 2025). Guidelines might also recognise that animal welfare science has a role in assessing AI’s impacts (Dawkins, 2021; Webber et al., 2022).

A further step would be to engage with and learn from nonhuman animals themselves to understand their preferences and participation in the avoidance of harmful AI and the creation of animal-friendly AI (Dawkins, 2004; Mancini, 2017). This could occur through careful attention to animal behaviour in relation to AI technology (Castelló, 2025; Meijer, 2019). It could also occur through listening to people who can legitimately and appropriately represent animal interests without their own conflicted and vested interests. Possible relevant parties could include respected animal advocacy organisations and animal welfare experts. Such organisations and experts already sit on official animal welfare standards making committees in some jurisdictions (Bromberg, 2024) and could be consulted in both the development and implementation of AI ethics guidelines as suitable representatives of animal interests. Such consultation and representation can also confer additional legitimacy on guidelines.

A further step to better ensure consideration of animal interests in AI ethics processes would be the creation of new bodies to act as representatives or guardians of animal interests in relation to datasets about animals and to AI models trained for purposes such as veterinary medicine, animal agriculture industries, biodiversity conservation, and other areas. Scholars of animal law and ethics have previously suggested that such bodies are needed to represent animal interests in relation to industry, environmental conservation, and legal regulation of animal welfare and the keeping of animals (Favre, 2000; Hadley, 2015).

It is well accepted in many jurisdictions that scientists who use animals for scientific research must have their research assessed by animal ethics committees to ensure that harm to animals is minimised or at least ethically justified, although the softness of these approaches is also criticised (Milford et al., 2025; Russell, 2011). Inspiration could be taken from these approaches to create bodies to act as guardians of animal datasets and machine learning models to ensure they are used for animals’ benefit and the avoidance of harm as much as possible.

AI ethics guidelines and codes could appreciate not only AI's potential to inflict both legal and illegal harms on animals but also its potential ‘for good’ (AI for Good Foundation, 2022; Taddeo & Floridi, 2018)—that is, for both reducing existing harms and exploitation and providing positive benefit for animals (Bossert, 2023; Coghlan & Parker, 2024). Consider, for example, AI that minimises animal suffering, fosters choice and autonomy (Mancini, 2017), enhances beneficial human-animal communication (Ryan & Bossert, 2024), and nourishes valuable human-animal bonds (Walsh, 2009). Animal-focused conservation organisations working on human animal communication, such as ‘translating’ whale song to be legible to humans, are currently working on creating ethics frameworks for such work, to ensure both the benefits are realised and potential misuse and harm avoided (Rodríguez-Garavito et al., 2025).

Ethical statements could recognise AI’s scope for finding new and creative ways of doing good for humans and animals alike. For example, as noted, AI might be used to replace some harmful animal experiments and to explore and promote replacements for animal products that cause the strict confinement and suffering of billions of animals (Singer, 2023). Integrated with technologies like robots and virtual reality, AI might also be used to create vivid experiences of wild animals that reduces the number of animals kept in restrictive and undignified zoo or theme park settings (Bossert, 2023; Gruen, 2014a).

Providing inspiring examples of AI’s possible positive impacts alongside illustrations of its capacity to compound existing animal suffering and create new sources of harm could be very useful additions to guidelines (Coghlan & Parker, 2023; Singer & Tse, 2022).

Ethics statements of values might also connect animal protection to relevant existing and familiar ‘higher-level’ principles which have hitherto been understood entirely human-centrically. Such principles include non-maleficence, beneficence, transparency, safety, explainability, and accountability (Jobin et al., 2019). An animal welfare or advocacy group could take on the task of specifying how such principles should be extended to animals and providing examples of their application that could be then adopted and adapted by relevant businesses, professional organisations, and civil society organisations.

It is worth remembering that statements of principles and values are not sufficient for delivering precise practical ethical guidance in specific contexts. AI will be deployed in many different domains, some with distinctive moral characters and requirements that must be accommodated (Kazim & Koshiyama, 2021; Mittelstadt, 2019). Higher-level moral principles can also conflict with each other and generate dilemmas. And because a ‘gap’ exists between general principles and concrete circumstances, moral principles must be specified and ‘operationalised’ (Hao, 2019; Lin, 2024; Morley et al., 2021; Morley et al., 2021; Munn, 2022; Prem, 2023; Whittlestone et al., 2019). This can require careful moral thought and judgement.

Although the higher-level nature of many AI ethics principles comes in for criticism, this sort of criticism has been previously considered and addressed in fields of practical ethics (Beauchamp & Childress, 2001; Seger, 2022). For example, the allegedly fatal ‘abstractness’ of certain principles, values, or virtues (like benevolence, justice, compassion, etc.) can instead underpin their power and usefulness. Nonetheless, thoughtfully translating these general norms to real-life cases and resolving resulting moral conflicts involving AI, animals, and humans—rather than merely ticking off checklists—will obviously be necessary. As Elizabeth Seger (2022, p. 6) writes:

[T]he most effective AI governance strategy is a dual approach; explicit rules and regulations should be buttressed by principle-based initiatives encouraging cultural change within AI research and development communities to deemphasize the cultural norms like optimization and scale that can be antagonistic to the goals of AI governance, and to promote values like beneficence and responsibility.

Ethics guidelines could, then, highlight important general principles, values, and virtues in relation to sentient nonhuman animals, while also noting that these guiding norms need to be developed, interpreted, and applied in specific contexts through moral dialogue, judgement, and internal procedures and processes.

## Conclusion

In this paper, we presented a moral case for including sentient animals in direct, explicit, and adequate ways in existing and future AI ethics guidelines produced by various companies, governments, NGOs, supranational and intergovernmental bodies, professional and academic organizations, and so on. By countering five key objections to including sentient animals as morally significant interest-holders in AI ethics guidelines, we argued that recognising the ethical need to protect animals and advance their interests in AI design and use is important and urgent. A central justifying reason in our case for including sentient animals in AI guidelines (and wider AI discourse) is their moral and social invisibility, which persists despite growing public concern about animal welfare.

Appropriately recognizing animals in AI ethics guidelines, while only one step among others required to protect and benefit them, could conceivably help stimulate much-needed debate about AIs good and bad effects on these morally significant sentient beings. Although it may seem overly optimistic to sceptics of ethics guidelines, such ethical statements and associated dialogue might have some welcome influence, however modest, on future development and regulation of AI. Our hope is that the argument developed in this paper can motivate organisations and guideline authors to thoughtfully include sentient animals in current and future AI ethics guidelines.

## Data Availability

No new data were generated or analysed in this research.

## Footnotes

1 Note that some ethics guidelines employ terms other than ‘AI’ that nonetheless also imply or encompass AI—e.g. ‘intelligent technology’, ‘autonomous machines’, etc.

2 Some scholars wonder whether current or future AI systems might themselves gain consciousness and morally relevant interests (Schwitzgebel, 2023). We leave this issue aside in this essay.

3 For lists of such guidelines see, e.g., https://inventory.algorithmwatch.org/, https://www.aiethicist.org/ai-principles (Ghoz & Hendawy, 2023), Accessed 17 November 2025.

4 Although some guidelines also outline more specific steps for making AI systems ethical (Prem, 2023).

5 G20 Maceió Ministerial Declaration on Digital Inclusion for All (Maceió, 13/09/2024) at https://g7g20-documents.org/database/document/2024-g20-brazil-sherpa-track-digital-economy-ministers-ministers-language-g20-dewg-maceio-ministerial-declaration. Accessed 19 September 2025.

6 Microsoft Responsible AI Standard, v2 (June 2022) at https://cdn-dynmedia-1.microsoft.com/is/content/microsoftcorp/microsoft/final/en-us/microsoft-brand/documents/Microsoft-Responsible-AI-Standard-General-Requirements.pdf?culture=en-us%26country=us. Accessed 19 September 2025.

7 Google AI, AI Principles: Our Approach to Building Beneficial AI (undated, but from about 2019) https://ai.google/static/documents/EN_US-AI-Principles.pdf; https://ai.google/responsibility/principles/. Accessed 19 September 2025.

8 Department of Industry, Science and Resources. (2024). *Australia’s Artificial Intelligence Ethics Principles*. https://Www.Industry.Gov.Au/Node/91877; and Department of Industry, Science and Resources. (2024). Voluntary AI Safety Standard. https://www.industry.gov.au/publications/voluntary-ai-safety-standard In the updated Guidance for AI Adoption released in October 2025, even the brief mention of ‘environmental’ responsibilities is missing: National Artificial Intelligence Centre, Department of Industry, Science and Resources. (2025). Guidance for AI Adoption. https://www.industry.gov.au/publications/guidance-for-ai-adoption. All accessed 27 October 2025.

9 OECD, Recommendation of the Council on Artificial Intelligence, OECD/LEGAL/0449 (22 May 2019, as amended 3 May 2024) at https://legalinstruments.oecd.org/en/instruments/OECD-LEGAL-0449. Accessed 19 September 2025.

10 UNESCO, Recommendation on the Ethics of Artificial Intelligence (23 November 2021) at https://unesdoc.unesco.org/ark:/48223/pf0000381137; Accessed 19 September 2025.

11 https://www.unesco.org/en/artificial-intelligence/recommendation-ethics. Accessed 31 October 2025.

12 UNESCO, Recommendation on the Ethics of Artificial Intelligence (23 November 2021) at https://unesdoc.unesco.org/ark:/48223/pf0000381137; Accessed 19 September 2025. At page 5.

13 Ibid, paragraph 17, page 19.

14 IEEE, Ethically Aligned Design: A Vision for Prioritizing Human Well-being with Autonomous and Intelligent Systems (First Addition), (undated), p. 65 at https://sagroups.ieee.org/global-initiative/wp-content/uploads/sites/542/2023/01/ead1e.pdf. Accessed 20 November 2025.

15 High-Level Expert Group on Artificial Intelligence (AI HLEG), Ethics Guidelines for Trustworthy Artificial Intelligence (8 April 2019, European Commission) p. 27 at https://ec.europa.eu/futurium/en/ai-alliance-consultation0.1.html. Accessed 19 September 2025.

16 See page 12 of that document.

17 EU AI Act (2024) at https://eur-lex.europa.eu/legal-content/EN/TXT/?uri=CELEX:32024R1689

18 Recital 1.

19 European Commission. (2025) The General-Purpose AI Code of Practice, p. 34. https://digital-strategy.ec.europa.eu/en/policies/contents-code-gpai. Accessed 25 October 2025. Personal communications with the AI for Animals group, now Sentient Futures, in October and November 2025 confirmed the process by which this occurred.

20 Montréal Declaration for a Responsible Development of Artificial Intelligence (2018) page 8 at https://declarationmontreal-iaresponsable.com/wp-content/uploads/2023/04/UdeM_Decl-IA-Resp_LA-Declaration-ENG_WEB_09-07-19.pdf. Accessed 19 September 2025.

21 Ibid. It is somewhat unclear whether ‘sentient beings’ in the document also refers to humans; further, the term ‘animals’ is not used under the Well-being principle. Still, the natural reading of ‘sentient beings’ is that it also refers to nonhuman animals.

22 It is a sufficient rather than necessary condition because there may be other reasons why an entity could have intrinsic moral value. We lack space to discuss this issue in any depth in this essay, though we shall briefly touch on it later. Our focus in this paper is on the appropriate recognition of sentient animals.

23 There are other possible grounds for intrinsic moral value in animals, such as merely being alive (P. W. Taylor, 2011). We concentrate on sentience (and preference possession) here because it is a more widely accepted ground of intrinsic value.

24 For brevity, we mostly refer simply to ‘sentient animals’ in this paper without always mentioning desires or preferences; but we should remember that some philosophers consider desires and preferences to be morally important.

25 Similar criticism targets ethics committees and ethics experts in tech companies (Bietti, 2021).

26 See https://blog.google/technology/ai/ai-principles. Google shifted course after Donald Trump’s 2024 re-election. Google’s new principles (https://ai.google/principles/) omit this and other commitments. Accessed 19 September 2025.

## References

[CR1] ABA. (2024, July 29). *ABA issues first ethics guidance on a lawyer’s use of AI tools*. American Bar Association News. https://www.americanbar.org/news/abanews/aba-news-archives/2024/07/aba-issues-first-ethics-guidance-ai-tools/

[CR2] Adams, C. J. (2000). *Sexual politics of meat: A feminist-Vegetarian Critical theory: A feminist-Vegetarian Critical theory*. Bloomsbury Publishing

[CR3] AHPRA. (2024). Meeting your professional obligations when using artificial intelligence in healthcare. *Australian Health Practitioner Regulation Agency*. https://www.ahpra.gov.au/Resources/Artificial-Intelligence-in-healthcare.aspx

[CR4] AI for Good Foundation. (2022). *About us-AI for good foundation*. https://ai4good.org/about-us/

[CR5] Antanaitis, R., Malašauskienė, D., Televičius, M., Juozaitienė, V., Žilinskas, H., & Baumgartner, W. (2020). Dynamic changes in progesterone concentration in cows’ Milk determined by the At-Line Milk Analysis system herd NavigatorTM. *Sensors*, *20*(18), Article 18. 10.3390/s20185020 10.3390/s20185020 PMC7570932 32899624

[CR6] Armstrong, S. J., & Botzler, R. G. (Eds.). (2017). *The animal ethics reader* (Third ed.). Routledge/Taylor & Francis Group

[CR7] Austin, J. L. (1975). *How to do things with words*. Harvard University Press

[CR8] Beauchamp, T. L., & Childress, J. F. (2001). *Principles of biomedical ethics*. Oxford University Press. 10.1136/jme.28.5.332-a

[CR9] Bender, E. M., & Hanna, A. (2025). *The AI Con: How to fight big tech’s hype and create the future we want*. Random House

[CR10] Bentham, J. (J. Burns, & H. Hart (Eds.). (1996). *The collected works of Jeremy Bentham: An introduction to the principles of morals and legislation*. Oxford University Press

[CR11] Bietti, E. (2021). From ethics washing to ethics bashing: A view on tech ethics from within moral Philosophy. *SSRN Electronic Journal*. 10.2139/ssrn.3914119

[CR12] Birch, J., Schnell, A. K., & Clayton, N. S. (2020). Dimensions of animal consciousness. *Trends in Cognitive Sciences*, *24*(10), 789–801. 10.1016/j.tics.2020.07.007 32830051 10.1016/j.tics.2020.07.007 PMC7116194

[CR13] Bos, J. M., Bovenkerk, B., Feindt, P. H., & van Dam, Y. K. (2018). The quantified animal: Precision Livestock farming and the ethical implications of objectification. *Food Ethics*, *2*(1), Article 1. 10.1007/s41055-018-00029-x

[CR14] Bossert, L. N. (2023). Benefitting nonhuman animals with AI: Why going Beyond “do No harm” is important. *Philosophy & Technology*, *36*(3), 57. 10.1007/s13347-023-00658-z

[CR15] Bossert, L. N., & Coeckelbergh, M. (2024). From MilkingBots to RoboDolphins: How AI changes human-animal relations and enables alienation towards animals. *Humanities and Social Sciences Communications*, *11*(1), 1–7. 10.1057/s41599-024-03441-3

[CR16] rennan, A., & Lo, N. Y. S. (2002). Environmental Ethics. In E. N. Zalta & U. Nodelman (Eds.), *The Stanford encyclopedia of philosophy (Summer 2024 Edition)*. https://plato.stanford.edu/archives/sum2024/entries/ethics-environmental/

[CR17] Bromberg, L. (2021). Numbing the pain or diffusing the pressure? The co-optation of PETA’s “naming and shaming” campaign against mulesing. *Law & Policy*, *43*(3), 285–313. 10.1111/lapo.12172

[CR18] Bromberg, L. (2024). *Applying a multi-species account of law and policy to evaluate the legitimacy of Australian farmed animal welfare standards*. PhD thesis, The University of Melbourne. https://hdl.handle.net/11343/355940

[CR19] Browning, H., & Birch, J. (2022). Animal sentience. *Philosophy Compass*, *17*(5), e12822. 10.1111/phc3.12822 35859762 10.1111/phc3.12822 PMC9285591

[CR20] Brownsword, R. (2022). *Rethinking Law, regulation, and technology*. Edward Elgar Publishing

[CR21] Burti, S., Banzato, T., Coghlan, S., Wodziniski, M., Bendazzoli, M., & Zotti, A. (2024). Artificial intelligence in veterinary diagnostic imaging: Perspectives and limitations. *Research in Veterinary Science*, *175*, 105317. 10.1016/j.rvsc.2024.105317 38843690 10.1016/j.rvsc.2024.105317

[CR22] Castelló, P. P. (2025). The fabric of zoodemocracy: A systemic approach to deliberative zoodemocracy. *Critical Review of International Social and Political Philosophy*, 1–26. 10.1080/13698230.2024.2437870

[CR23] Cellard, L., Parker, C., & Haines, F. (2025). Beyond AI as an environmental pharmakon: Principles for reopening the problem-space of machine learning’s carbon footprint. *Environment and Planning E*, *25148486251332087*. 10.1177/25148486251332087

[CR24] Centers for Disease Control and Prevention. (2022, February 7). *One Health Basics*. https://www.cdc.gov/onehealth/basics/index.html

[CR25] Charlton, G., Falzon, G., Shepley, A., Fleming, P. J. S., Ballard, G., Meek, P. D., Charlton, G., Falzon, G., Shepley, A., Fleming, P. J. S., Ballard, G., & Meek, P. D. (2023). The sentinel bait station: An automated, intelligent design pest animal baiting system. *Wildlife Research*. 10.1071/WR22183

[CR26] Coghlan, S. (2025). Nonhuman animal dignity. *Philosophy Compass*, *20*(4), e70035. 10.1111/phc3.70035

[CR27] Coghlan, S., & Cardilini, A. P. A. (2022). A critical review of the compassionate conservation debate. *Conservation Biology*, *36*(1), e13760. 10.1111/cobi.13760 34057240 10.1111/cobi.13760

[CR28] Coghlan, S., & Parker, C. (2023). Harm to nonhuman animals from AI: A systematic account and framework. *Philosophy & Technology*, *36*(2), 25. 10.1007/s13347-023-00627-6

[CR29] Coghlan, S., & Parker, C. (2024). Helping and not harming animals with AI. *Philosophy & Technology*, *37*(1), 20. 10.1007/s13347-024-00712-4

[CR30] Coghlan, S., & Quinn, T. (2023). Ethics of using artificial intelligence (AI) in veterinary medicine. *AI & SOCIETY*. 10.1007/s00146-023-01686-1

[CR31] Cornish, A., Raubenheimer, D., & McGreevy, P. (2016). What we know about the public’s level of concern for farm animal welfare in food production in developed countries. *Animals*, *6*(11), Article 11. 10.3390/ani6110074 10.3390/ani6110074 PMC5126776 27854336

[CR32] Corrêa, N. K., De Oliveira, N., & Massmann, D. (2022). On the efficiency of ethics as a governing tool for Artificial Intelligence (No. arXiv: 2210.15289). arXiv. 10.48550/arXiv.2210.15289

[CR33] Corrêa, N. K., Galvão, C., Santos, J. W., Del Pino, C., Pinto, E. P., Barbosa, C., Massmann, D., Mambrini, R., Galvão, L., Terem, E., & de Oliveira, N. (2023). Worldwide AI ethics: A review of 200 guidelines and recommendations for AI governance. *Patterns*, *4*(10), 100857. 10.1016/j.patter.2023.100857 37876898 10.1016/j.patter.2023.100857 PMC10591196

[CR34] Crary, A., & Gruen, L. (2022). *Animal crisis: A new Critical theory*. Polity Press

[CR35] Crofts, P., & van Rijswijk, H. (2020). Negotiating “evil”: Google, project maven and the corporate form. *Law, Technology and Humans*, *2*(1), 75–90. 10.3316/informit.125958904331884

[CR36] Curtin, S. (2009). Wildlife tourism: The intangible, psychological benefits of human-wildlife encounters. *Current Issues in Tourism*, *12*(5–6), 451–474. 10.1080/13683500903042857

[CR37] Danks, D. (2022). Digital ethics as translational ethics. In I. Vasiliu-Feltes, & J. Thomason (Eds.), *Advances in human and social aspects of technology* (pp. 1–15). IGI Global. 10.4018/978-1-7998-8467-5.ch001

[CR38] Dawkins, M. S. (2004). Using behaviour to assess animal welfare. *Animal Welfare*, *13*(S1), S3–S7. 10.1017/S0962728600014317

[CR39] Dawkins, M. S. (2021). Does Smart farming improve or damage animal welfare? Technology and what animals want. *Frontiers in Animal Science*, *2*, 38. 10.3389/fanim.2021.736536

[CR40] DeGrazia, D. (1996). *Taking animals seriously: Mental life and moral status*. Cambridge University Press

[CR41] den Bergh, J., & Deschoolmeester, D. (2010). Ethical decision making in ICT: Discussing the impact of an ethical code of conduct. *Communications of the IBIMA*, 1–11. 10.5171/2010.127497

[CR42] Dhont, K., Hodson, G., & Leite, A. C. (2016). Common ideological roots of speciesism and generalized ethnic prejudice: The social dominance human-animal relations Model (SD-HARM). *European Journal of Personality*, *30*(6), 507–522. 10.1002/per.2069

[CR43] Dhont, K., Piazza, J., & Hodson, G. (2021). The role of meat appetite in willfully disregarding factory farming as a pandemic catalyst risk. *Appetite*, *164*, 105279. 10.1016/j.appet.2021.105279 33930493 10.1016/j.appet.2021.105279 PMC9756515

[CR44] Donaldson, S., & Kymlicka, W. (2011). *Zoopolis: A political theory of animal rights*. Oxford University Press

[CR45] Donovan, J., & Adams, C. J. (Eds.). (2007). *The feminist care tradition in animal ethics: A reader*. Columbia University Press

[CR46] Džermeikaitė, K., Bačėninaitė, D., & Antanaitis, R. (2023). Innovations in cattle farming: Application of innovative technologies and sensors in the diagnosis of diseases. *Animals*, *13*(5), Article 5. 10.3390/ani13050780 10.3390/ani13050780 PMC10000156 36899637

[CR47] Eisen, M. B., & Brown, P. O. (2022). Rapid global phaseout of animal agriculture has the potential to stabilize greenhouse gas levels for 30 years and offset 68 percent of CO2 emissions this century. *PLOS Climate*, *1*(2), e0000010. 10.1371/journal.pclm.0000010

[CR48] Eubanks, V. (2018). *Automating inequality: How high-tech tools profile, police, and punish the poor*. St. Martin’s Press

[CR49] Favre, D. (2000). Equitable self-ownership for animals. *Duke Law Journal*, *50*, 473–502. https://scholarship.law.duke.edu/dlj/vol50/iss2/2

[CR50] Ferrell, O. C., & Ferrell, L. (2024). Building a better world: The role of AI ethics and Social responsibility. *Journal of Macromarketing*, *44*(4), 928–935. 10.1177/02761467241285793

[CR51] Fraser, D. (2012). A “practical” Ethic for animals. *Journal of Agricultural and Environmental Ethics*, *25*(5), Article 5. 10.1007/s10806-011-9353-z

[CR52] Gangwal, A., & Lavecchia, A. (2025). Artificial intelligence in preclinical research: Enhancing digital twins and organ-on-chip to reduce animal testing. *Drug Discovery Today*, *30*(5), 104360. 10.1016/j.drudis.2025.104360 40252989 10.1016/j.drudis.2025.104360

[CR53] Ghose, S., Tse, Y. F., Rasaee, K., Sebo, J., & Singer, P. (2024). The case for animal-friendly AI (No. arXiv: 2403.01199). arXiv. 10.48550/arXiv.2403.01199

[CR54] Ghoz, L., & Hendawy, M. (2023). An inventory of AI ethics: Analyzing 100 documents. *MSA Engineering Journal*, *2*(2), 647–675. 10.21608/msaeng.2023.291907

[CR55] Gibb, R., Redding, D. W., Chin, K. Q., Donnelly, C. A., Blackburn, T. M., Newbold, T., & Jones, K. E. (2020). Zoonotic host diversity increases in human-dominated ecosystems. *Nature*, *584*(7821), 398–402. 10.1038/s41586-020-2562-8 32759999 10.1038/s41586-020-2562-8

[CR56] Gießler, S., & Hass, L. (2020, April). *Ethics between business lingo and politics: Why bother? AI ethics guidelines global inventory*. https://inventory.algorithmwatch.org/about

[CR57] Goodpaster, K. (1978). On being morally considerable. *The Journal of Philosophy*, *75*(6), 308–325. 10.2307/2025709

[CR58] Griffin, R. (2024). Codes of Conduct in eu digital regulation and AI policy: The potential and risks of soft Law tools. In K. Prifti, E. Demir, J. Krämer, K. Heine, & E. Stamhuis (Eds.), *Digital governance: Confronting the challenges posed by artificial intelligence* (pp. 253–271). T.M.C. Asser Press. 10.1007/978-94-6265-639-0_12

[CR59] Gruen, L. (2014a). Dignity, Captivity, and an ethics of sight. In L. Gruen (Ed.), *The ethics of Captivity* (pp. 231–247). Oxford University Press. 10.1093/acprof:oso/9780199977994.003.0015

[CR60] Gruen, L. (2014b). *Entangled empathy: An alternative Ethic for our relationships with animals* (1st ed.). Lantern Books

[CR61] Gruen, L., & Monsó, S. (2024). The moral status of animals. In E. N. Zalta, & U. Nodelman (Eds.), *The stanford encyclopedia of philosophy*. Metaphysics Research Lab, Stanford University. https://plato.stanford.edu/archives/fall2024/entries/moral-animal/

[CR62] Hacker, P. (2024). Sustainable AI regulation. *Common Market Law Review*, *61*(2), 345–386. 10.54648/cola2024025

[CR63] Hadley, J. (2015). *Animal property rights: A theory of habitat rights for wild animals*. Lexington Books

[CR64] Hagendorff, T. (2020). The ethics of AI ethics: An evaluation of guidelines. *Minds and Machines*, *30*(1), 99–120. 10.1007/s11023-020-09517-8

[CR65] Hagendorff, T. (2021). Blind spots in AI ethics. *AI and Ethics*. 10.1007/s43681-021-00122-8

[CR66] Hagendorff, T. (2022). Blind spots in AI ethics. *AI and Ethics*, *2*(4), 851–867. 10.1007/s43681-021-00122-8

[CR67] Hagendorff, T., Bossert, L., Fai, T. Y., & Singer, P. (2022). Speciesist bias in AI - How AI applications perpetuate discrimination and unfair outcomes against animals. arXiv: 2202.10848 [Cs]. http://arxiv.org/abs/2202.10848

[CR68] Hao, K. (2019). In 2020, let’s stop AI ethics-washing and actually do something. *Mit technology review*. https://www.technologyreview.com/2019/12/27/57/ai-ethics-washing-time-to-act/

[CR69] Hass, L., & Gießler, S. (2020). In the realm of paper tigers - exploring the failings of AI ethics guidelines. *AlgorithmWatch*. https://algorithmwatch.org/en/ai-ethics-guidelines-inventory-upgrade-2020/

[CR70] Hayek, M. N. (2022). The infectious disease trap of animal agriculture. *Science Advances*, *8*(44), eadd6681. 10.1126/sciadv.add6681 36322670 10.1126/sciadv.add6681 PMC9629715

[CR71] Héder, M. (2020). A criticism of AI ethics guidelines. *Információs Társadalom*, *20*(4), 57–73. 10.22503/inftars.XX.2020.4.5

[CR72] International Research Center for AI Ethics and Governance. (2022, January 10). *Artificial intelligence for children: Beijing Principles*. International Research Center for AI Ethics and Governance. https://ai-ethics-and-governance.institute/artificial-intelligence-for-children-beijing-principles/

[CR73] Jaworska, A., & Tannenbaum, J. (2021 2021). The grounds of moral status. In E. N. Zalta (Ed.), *The stanford encyclopedia of philosophy*. Metaphysics Research Lab, Stanford University. https://plato.stanford.edu/archives/spr2021/entries/grounds-moral-status/ (Spring

[CR74] Jia, C. (2022, December 14). Tech heralds clean, productive future for pig farms. *China Daily*.//global.chinadaily.com.cn/a/202212/14/WS639929c2a31057c47eba44da.html

[CR75] Jobin, A., Ienca, M., & Vayena, E. (2019). The global landscape of AI ethics guidelines. *Nature Machine Intelligence*, *1*(9), 389–399. 10.1038/s42256-019-0088-2

[CR76] Kant, I. (2002). *Groundwork for the Metaphysics of Morals* (A. W. Wood, Ed.). Yale University Press.

[CR77] Karthick, G. S., Sridhar, M., & Pankajavalli, P. B. (2020). Internet of things in animal healthcare (IoTAH): Review of recent advancements in architecture, sensing technologies and real-time monitoring. *SN Computer Science*, *1*(5), 301. 10.1007/s42979-020-00310-z

[CR78] Kayser-Bril, N. (2019). Ethical guidelines issued by engineers’ organization fail to gain traction. *AlgorithmWatch*. https://algorithmwatch.org/en/ieee-ethically-aligned-design-guidelines-fail-to-gain-traction/

[CR79] Kazim, E., & Koshiyama, A. S. (2021). A high-level overview of AI ethics. *Patterns*, *2*(9). 10.1016/j.patter.2021.100314 10.1016/j.patter.2021.100314 PMC8441585 34553166

[CR80] Korsgaard, C. M. (1996). *The sources of Normativity*. Cambridge University Press

[CR81] Kotzmann, J. (2023). Sentience and intrinsic worth as a pluralist Foundation for fundamental animal rights. *Oxford Journal of Legal Studies*, *43*(2), 405–428. 10.1093/ojls/gqad003 37287904 10.1093/ojls/gqad003 PMC10243921

[CR82] Kymlicka, W. (2018). Human rights without human supremacism. *Canadian Journal of Philosophy*, *48*(6), 763–792. 10.1080/00455091.2017.1386481

[CR83] Larsson, S. (2020). On the governance of artificial intelligence through ethics guidelines. *Asian Journal of Law and Society*, *7*(3), 437–451. 10.1017/als.2020.19

[CR84] Lauer, D. (2021). You cannot have AI ethics without ethics. *AI and Ethics*, *1*(1), 21–25. 10.1007/s43681-020-00013-4

[CR85] Leahy, M. P. T. (2005). *Against Liberation: Putting animals in perspective*. Routledge. 10.4324/9780203981238

[CR86] Lederman, Z. (2024). Towards an ethical analysis of research in one health (EAROH). *Journal of Bioethical Inquiry*. 10.1007/s11673-024-10406-3 10.1007/s11673-024-10406-3 PMC12263795 39671167

[CR87] Levy, N. (2021). Virtue signalling is virtuous. *Synthese*, *198*(10), 9545–9562. 10.1007/s11229-020-02653-9

[CR88] Levy, R. (2022, December 6). Musk’s neuralink faces federal inquiry after killing 1, 500 animals in testing. *The guardian*. https://www.theguardian.com/technology/2022/dec/05/neuralink-animal-testing-elon-musk-investigation

[CR89] Lin, Z. (2024). Beyond principlism: Practical strategies for ethical AI use in research practices. *AI and Ethics*. 10.1007/s43681-024-00585-5

[CR90] Luccioni, A. S., & Rolnick, D. (2023). Bugs in the data: How ImageNet misrepresents biodiversity. *Proceedings of the AAAI Conference on Artificial Intelligence*, *37*(12), Article 12. 10.1609/aaai.v37i12.26682

[CR91] Mahfuz, S., Mun, H.-S., Dilawar, M. A., & Yang, C.-J. (2022). Applications of Smart technology as a sustainable strategy in modern swine farming. *Sustainability*, *14*(5), Article 5. 10.3390/su14052607

[CR92] Mancini, C. (2017). Towards an animal-centred ethics for animal-Computer Interaction. *International Journal of Human-Computer Studies*, *98*, 221–233. 10.1016/j.ijhcs.2016.04.008

[CR93] Mancini, C. (2024). Responsible ACI: Expanding the influence of animal-Computer Interaction. In *Proceedings of the tenth international conference on animal-computer interaction* (pp. 1–9). 10.1145/3637882.3637895

[CR94] May, T. (2014). Moral individualism, moral relationalism, and obligations to non-human animals. *Journal of Applied Philosophy*, *31*(2), 155–168. 10.1111/japp.12055

[CR95] McNamara, A., Smith, J., & Murphy-Hill, E. (2018). Does ACM’s code of ethics change ethical decision making in software development? In *Proceedings of the 2018 26th ACM joint meeting on Europeansoftware engineering conference and symposium on the foundations of software engineering* (pp. 729–733). 10.1145/3236024.3264833

[CR96] Meijer, E. (2019). *When animals speak: Toward an interspecies Democracy*. NYU Press

[CR97] Mellor, D. (2017). Operational details of the five domains model and its key applications to the assessment and management of animal welfare. *Animals*, *7*(12), 60. 10.3390/ani7080060 28792485 10.3390/ani7080060 PMC5575572

[CR98] Midgley, M. (1983). *Animals and why they matter*. Penguin Books

[CR99] Milford, A., Clercq, E. D., Louis-Maerten, E., Geneviève, L. D., & Elger, B. S. (2025). How animal ethics committees make decisions - a scoping review of empirical studies. *PLoS One*, *20*(3), e0318570. 10.1371/journal.pone.0318570 40096023 10.1371/journal.pone.0318570 PMC11913294

[CR100] Mittelstadt, B. (2019). Principles alone cannot guarantee ethical AI. *Nature Machine Intelligence*, *1*(11), 501–507. 10.1038/s42256-019-0114-4

[CR101] Morley, J., Elhalal, A., Garcia, F., Kinsey, L., Mökander, J., & Floridi, L. (2021). Ethics as a service: A pragmatic operationalisation of AI ethics. *Minds and Machines*, *31*(2), 239–256. 10.1007/s11023-021-09563-w 34720418 10.1007/s11023-021-09563-w PMC8550007

[CR102] Morley, J., Kinsey, L., Elhalal, A., Garcia, F., Ziosi, M., & Floridi, L. (2021). Operationalising AI ethics: Barriers, enablers and next steps. *AI & SOCIETY*. 10.1007/s00146-021-01308-8

[CR103] Morrison, J. (2014). *The Social license: How to keep your organization legitimate*. Palgrave Macmillan

[CR104] Munn, L. (2022). The uselessness of AI ethics. *AI and Ethics*. 10.1007/s43681-022-00209-w

[CR105] Nandutu, I., Atemkeng, M., & Okouma, P. (2023). Integrating AI ethics in wildlife conservation AI systems in South Africa: A review, challenges, and future research agenda. *AI & SOCIETY*, *38*, 245–257. 10.1007/s00146-021-01285-y

[CR106] Napolitano Ferreira, M., Ellio, W., Golden Kroner, R., Kinnaird, M. F., Prist, P. R., Valdujo, P., & Vale, M. M. (2021). Drivers and causes of zoonotic diseases: An overview. *Parks*, *27*, 15–24. 10.2305/IUCN.CH.2021.PARKS-27-SIMNF.en

[CR107] Narayanan, A., & Kapoor, S. (2024). *AI snake oil: What Artificial Intelligence can do, What it Can’t, and How to tell the difference*. Princeton University Press

[CR108] Neethirajan, S. (2021). Ethics of digital animal farming. *Preprints*, 2021070368. 10.20944/preprints202107.0368.v1

[CR109] Norman, W. (2011). Business ethics as self-regulation: Why principles that ground regulations should be used to ground beyond-compliance norms as well. *Journal of Business Ethics*, *102*(1), 43–57. 10.1007/s10551-011-1193-2

[CR110] Nussbaum, M. C. (2006). *Frontiers of justice: Disability, nationality, species membership*. Belknap Press

[CR111] Nussbaum, M. C. (2023). *Justice for animals: Our collective responsibility*. Simon and Schuster

[CR112] O’Neil, C. (2016). *Weapons of math destruction: How Big Data increases inequality and threatens Democracy*. Crown

[CR113] Ochigame, R. (2022). The invention of “ethical AI”: How big tech manipulates academia to avoid regulation. In T. Phan, J. Goldenfein, D. Kuch, & M. Mann (Eds.), *Economies of virtue - the circulation of “ethics” in AI* (pp. 49–56). Institute of Network Cultures. https://mediarep.org/handle/doc/20441

[CR114] Owe, A., & Baum, S. D. (2021). Moral consideration of nonhumans in the ethics of artificial intelligence. *AI and Ethics*, *1*, 517–528. 10.1007/s43681-021-00065-0

[CR115] Palmer, C. (2010). *Animal ethics in context*. Columbia University Press

[CR116] Parker, C. (2002). *The open corporation: Effective self-regulation and Democracy*. Cambridge University Press

[CR117] Phan, T., Goldenfein, J., Mann, M., & Kuch, D. (2022). Economies of virtue: The circulation of ‘ethics’ in Big tech. *Science as Culture*, *31*(1), 121–135. 10.1080/09505431.2021.1990875

[CR118] Pinillos, R. G., Appleby, M. C., Manteca, X., Scott-Park, F., Smith, C., & Velarde, A. (2016). One welfare-a platform for improving human and animal welfare. *Veterinary Record*, *179*(16), 412–413 27770094 10.1136/vr.i5470

[CR119] Pluhar, E. B. (1987). The personhood view and the argument from marginal cases. *Philosophica*, *39*(1), 23–38. 10.21825/philosophica.82493

[CR120] Png, M.-T. (2022). At the tensions of south and north: Critical roles of global south stakeholders in AI governance. *Proceedings of the 2022 ACM Conference on Fairness, Accountability, and Transparency*, 1434–1445 10.1145/3531146.3533200

[CR121] Prem, E. (2023). From ethical AI frameworks to tools: A review of approaches. *AI and Ethics*, *3*(3), 699–716. 10.1007/s43681-023-00258-9

[CR122] Pust, J. (2024). Intuition. In E. N. Zalta & U. Nodelman (Eds.), *The Stanford encyclopedia of philosophy* (Fall 2024). Metaphysics Research Lab, Stanford University. https://plato.stanford.edu/archives/fall2024/entries/intuition/

[CR123] Rees, C., & Müller, B. (2023). All that glitters is not gold: Trustworthy and ethical AI principles. *AI and Ethics*, *3*(4), 1241–1254. 10.1007/s43681-022-00232-x 10.1007/s43681-022-00232-x PMC9667859 36406882

[CR124] Regan, T. (2004). *The case for animal rights*. University of California Press

[CR125] Rességuier, A., & Rodrigues, R. (2020). AI ethics should not remain toothless! A call to bring back the teeth of ethics. *Big Data & Society*, *7*(2), 2053951720942541. 10.1177/2053951720942541

[CR126] Rickards, H., Read, J. L., Johnson, C. N., Jones, M. E., Pauza, M. D., Bentley, J., Sculthorpe, A., Humphrey, M., Hamer, R., Rickards, H., Read, J. L., Johnson, C. N., Jones, M. E., Pauza, M. D., Bentley, J., Sculthorpe, A., Humphrey, M., & Hamer, R. (2022). Is the Felixer cat control device safe for marsupial carnivores? *Wildlife Research*, *50*(5), 356–365. 10.1071/WR21175

[CR127] Rigley, E., Chapman, A., Evers, C., & McNeill, W. (2023). Anthropocentrism and environmental wellbeing in AI ethics standards: A scoping review and discussion. *AI*, *4*(4), Article 4. 10.3390/ai4040043

[CR128] Rodríguez-Garavito, C., Gruber, D. F., Nemeth, A. O., & Begus, G. (2025). What if we understood what animals are saying? The legal impact of AI-assisted studies of animal communication. *SSRN scholarly paper No. 5165527*. Social Science Research Network. 10.2139/ssrn.5165527

[CR129] Russell, D. (2011). Why animal ethics committees don’t work. *Between the Species*, *15*(1), 127–142. 10.15368/bts.2012v15n1.1

[CR130] Ryan, H. (2022). *Elon Musk’s neuralink confirms monkeys died in project, denies animal cruelty claims*. CNN Business. https://www.cnn.com/2022/02/17/business/elon-musk-neuralink-animal-cruelty-intl-scli/index.html

[CR131] Ryan, M. (2019). Ethics of using AI and Big Data in agriculture: The case of a large agriculture multinational. *The ORBIT Journal*, *2*(2), 1–27. 10.29297/orbit.v2i2.109

[CR132] Ryan, M. (2022). The social and ethical impacts of artificial intelligence in agriculture: Mapping the agricultural AI literature. *AI & SOCIETY*. 10.1007/s00146-021-01377-9

[CR133] Ryan, M., & Bossert, L. N. (2024). Dr. Doolittle uses AI: Ethical challenges of trying to speak whale. *Biological Conservation*, *295*, 110648. 10.1016/j.biocon.2024.110648

[CR134] Ryan, M., & Stahl, B. C. (2021). Artificial intelligence ethics guidelines for developers and users: Clarifying their content and normative implications. *Journal of Information, Communication and Ethics in Society*, *19*(1), 61–86. 10.1108/JICES-12-2019-0138

[CR135] Schiff, D., Biddle, J., Borenstein, J., & Laas, K. (2020). What’s next for AI ethics, policy, and governance? A global overview. In *Proceedings of the AAAI/ACM conference on AI, ethics, and society* (pp. 153–158). 10.1145/3375627.3375804

[CR136] Schwitzgebel, E. (2023). AI systems must not confuse users about their sentience or moral status. *Patterns*, *4*(8). 10.1016/j.patter.2023.100818 10.1016/j.patter.2023.100818 PMC10436038 37602213

[CR137] Seger, E. (2022). In defence of principlism in AI ethics and governance. *Philosophy & Technology*, *35*(2), 1–7. 10.1007/s13347-022-00538-y

[CR138] Serpell, J. (1996). *In the company of animals: A study of human-animal relationships*. Cambridge University Press

[CR139] Simoneau-Gilbert, V., & Birch, J. (2024, March 22). The dangers of AI farming. *Aeon*. https://aeon.co/essays/how-to-reduce-the-ethical-dangers-of-ai-assisted-farming

[CR140] Sinclair, M., Lee, N. Y. P., Hötzel, M. J., de Luna, M. C. T., Sharma, A., Idris, M., Derkley, T., Li, C., Islam, M. A., Iyasere, O. S., Navarro, G., Ahmed, A. A., Khruapradab, C., Curry, M., Burns, G. L., & Marchant, J. N. (2022). International perceptions of animals and the importance of their welfare. *Frontiers in Animal Science*, *3*, 960379. 10.3389/fanim.2022.960379

[CR141] Singer, P. (Ed.). (2006). *In defense of animals: The second wave*. Blackwell Publishing

[CR142] Singer, P. (2023). *Animal Liberation Now: The definitive Classic renewed*. Harper Perennial

[CR143] Singer, P., & Tse, Y. F. (2022). AI ethics: The case for including animals. *AI and Ethics*. 10.1007/s43681-022-00187-z

[CR144] Singh, D., Singh, R., Gehlot, A., Akram, S. V., Priyadarshi, N., & Twala, B. (2022). An imperative role of digitalization in monitoring Cattle health for sustainability. *Electronics*, *11*(17), Article 17. 10.3390/electronics11172702

[CR145] Sparrow, R., & Howard, M. (2021). Robots in agriculture: Prospects, impacts, ethics, and policy. *Precision Agriculture*, *22*(3), 818–833. 10.1007/s11119-020-09757-9

[CR146] Sparrow, R., Howard, M., & Degeling, C. (2021). Managing the risks of artificial intelligence in agriculture. *NJAS: Impact in Agricultural and Life Sciences*, *93*(1), 172–196. 10.1080/27685241.2021.2008777

[CR147] St. Pierre, S. R., Darwin, E. C., Adil, D., Aviles, M. C., Date, A., Dunne, R. A., Lall, Y., Parra Vallecillo, M., Perez Medina, V. A., Linka, K., Levenston, M. E., & Kuhl, E. (2024). The mechanical and sensory signature of plant-based and animal meat. *NPJ Science of Food*, *8*(1), 94. 10.1038/s41538-024-00330-6 39548076 10.1038/s41538-024-00330-6 PMC11568319

[CR148] Staff, G. (2022, November 25). China’s 26-storey pig skyscraper ready to slaughter 1 million pigs a year. *The Guardian*. https://www.theguardian.com/environment/2022/nov/25/chinas-26-storey-pig-skyscraper-ready-to-produce-1-million-pigs-a-year

[CR149] Steinfeld, H., Gerber, P., Wassenaar, T. D., Castel, V., & De Haan, C. (2006). *Livestock’s long shadow: Environmental issues and options*. Food and Agriculture Organisation

[CR150] Taddeo, M., & Floridi, L. (2018). How AI can be a force for good. *Science*, *361*(6404), 751–752. 10.1126/science.aat5991 30139858 10.1126/science.aat5991

[CR151] Tasioulas, J. (2022). Artificial Intelligence, humanistic ethics. *Artificial Intelligence*, *151*(2), 232–243. 10.1162/daed_a_01912

[CR152] Taylor, N. (1999). Whither rights? Animal rights and the rise of new welfarism. *Animal Issues*, *3*(1), 27–42

[CR153] Taylor, P. W. (2011). *Respect for nature: A theory of environmental ethics*. Princeton University Press

[CR154] Tosi, J., & Warmke, B. (2016). Moral grandstanding. *Philosophy & Public Affairs*, *44*(3), 197–217. 10.1111/papa.12075

[CR155] Tovey, H. (2003). Theorising nature and society in sociology: The invisibility of animals. *Sociologia Ruralis*, *43*(3), 196–215. 10.1111/1467-9523.00241

[CR156] Tuyttens, F. A. M., Molento, C. F. M., & Benaissa, S. (2022). Twelve threats of Precision Livestock farming (PLF) for animal welfare. *Frontiers in Veterinary Science*, *9*. https://www.frontiersin.org/articles/10.3389/fvets.2022.889623 10.3389/fvets.2022.889623 PMC9186058 35692299

[CR157] Vinnari, E., & Vinnari, M. (2022). Making the invisibles visible: Including animals in sustainability (and) accounting. *Critical Perspectives on Accounting*, *82*, 102324. 10.1016/j.cpa.2021.102324

[CR158] Walsh, F. (2009). Human-animal bonds I: The relational significance of companion animals. *Family Process*, *48*(4), 462–480. 10.1111/j.1545-5300.2009.01296.x 19930433 10.1111/j.1545-5300.2009.01296.x

[CR159] Webber, S., Cobb, M. L., & Coe, J. (2022). Welfare through competence: A framework for animal-centric technology design. *Frontiers in Veterinary Science*, *9*(885973), 1–20. 10.3389/fvets.2022.885973 10.3389/fvets.2022.885973 PMC9280685 35847650

[CR160] Werkheiser, I. (2018). Precision Livestock farming and farmers’ duties to livestock. *Journal of Agricultural and Environmental Ethics*, *31*(2), Article 2. 10.1007/s10806-018-9720-0

[CR161] Whittlestone, J., Nyrup, R., Alexandrova, A., & Cave, S. (2019). The role and limits of principles in AI ethics: Towards a focus on tensions. In *Proceedings of the 2019 AAAI/ACM conference on AI, ethics, and society* (pp. 195–200). 10.1145/3306618.3314289

[CR162] Williams, N., Hemsworth, L., Chaplin, S., Shephard, R., & Fisher, A. (2024). Are there risk factors commonly observed on Australian farms where the welfare of livestock is poor? *Animal Welfare*, *33*, e34. 10.1017/awf.2024.27 39315351 10.1017/awf.2024.27 PMC11418073

[CR163] Wurtz, K., Camerlink, I., D’Eath, R. B., Fernández, A. P., Norton, T., Steibel, J., & Siegford, J. (2019). Recording behaviour of indoor-housed farm animals automatically using machine vision technology: A systematic review. *PLoS One*, *14*(12), e0226669. 10.1371/journal.pone.0226669 31869364 10.1371/journal.pone.0226669 PMC6927615

[CR164] Yadin, S. (2025). The hidden nature of regulation. *SSRN scholarly paper No. 5211248*. Social Science Research Network. 10.2139/ssrn.5211248

[CR165] Yang, F., Goldenfein, J., & Nickels, K. (2024). GenAI concepts. *ADM+S centre*. https://www.admscentre.org.au/genai-concepts/

[CR166] Ziesche, S. (2021). AI ethics and value alignment for nonhuman animals. *Philosophies*, *6*(2), Article 2. 10.3390/philosophies6020031

[CR167] Zinsstag, J., Schelling, E., Waltner-Toews, D., & Tanner, M. (2011). From “one medicine” to “one health” and systemic approaches to health and well-being. *Preventive Veterinary Medicine*, *101*(3), 148–156. 10.1016/j.prevetmed.2010.07.003 20832879 10.1016/j.prevetmed.2010.07.003 PMC3145159

